# Spontaneous retroperitoneal haemorrhage after pulmonary endarterectomy surgery

**DOI:** 10.1186/s13019-024-02726-7

**Published:** 2024-05-18

**Authors:** Aabha Divya, Alicia Chia, David Jenkins

**Affiliations:** Cardiothoracic Surgery, Royal Papworth Hospital NHS Trust, Cambridge, CB2 0AY UK

**Keywords:** Pulmonary endarterectomy, Spontaneous retroperitoneal haemorrhage, Anticoagulation

## Abstract

Spontaneous retroperitoneal hematoma (SRH) is a rare complication of anticoagulation therapy. Presentation may vary from limb paresis to hypovolemic shock due to blood loss. The optimal treatment is controversial. It can be managed conservatively or surgically. We report a case of a 73-year-old man presenting with progressively worsening abdominal pain and severe pain radiating to his left lower limb twenty-five days after his pulmonary endarterectomy (PEA) surgery. He was on anticoagulation per our institutional protocol for PEA patients. Investigations revealed a large, spontaneously occurring iliopsoas hematoma. Our patient was treated conservatively, and the SRH stabilised.

## Introduction

Haemorrhage resulting in a spontaneous retroperitoneal hematoma (SRH) is a rare entity with unknown aetiology. Clinical trials involving anticoagulant drugs classify this occurrence as “major bleeding,” with an incidence ranging from 0.6 to 6.6% and a mortality rate of approximately 20% [1, 2]. Risk factors include anticoagulation, elderly age, and patients undergoing dialysis [3]. Clinical presentation is highly variable; hence, diagnosis can be challenging. SRH can be potentially fatal unless it is diagnosed and treated in time. The optimal treatment is controversial. It can be managed conservatively or surgically. We report a case of a 73-year-old man presenting with progressively worsening abdominal pain and severe pain radiating to his left lower limb twenty-five days after his pulmonary endarterectomy (PEA) surgery. He was on anticoagulation per our institutional protocol for PEA patients. Investigations revealed a large, spontaneously occurring iliopsoas hematoma. Our patient was treated conservatively, and the SRH stabilised.

## Case report

**Fig. 1:**
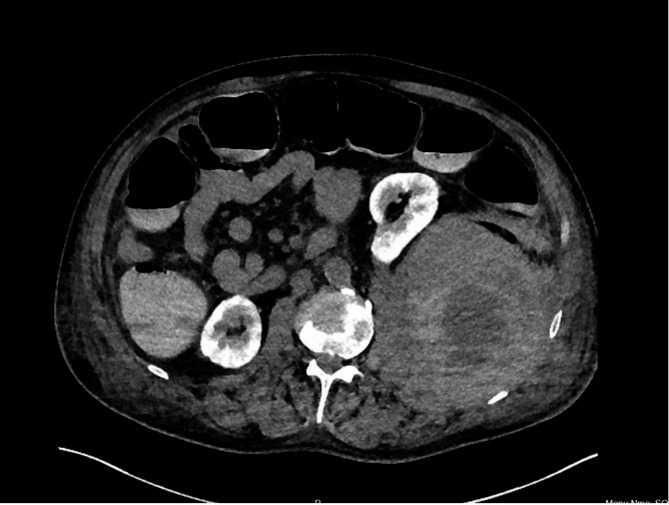
Computed tomography scan of abdomen showing retroperitoneal haematoma (red arrow) involving the left psoas muscle, with displacement of the left kidney (yellow arrow) anteriorly

Our patient is a 73-year-old man referred to the Royal Papworth Hospital, Cambridge, United Kingdom, for chronic thromboembolic pulmonary hypertension (CTEPH). He had left-sided deep venous thrombosis and pulmonary embolism diagnosed in January 2021, for which he had been initiated on anticoagulation with Rivaroxaban 20 mg once daily. Other significant past medical issues include coronary artery disease, obstructive sleep apnoea treated with continuous positive airway pressure therapy (CPAP), chronic idiopathic lymphoedema and extensive granulomatous lymphadenopathy from sarcoidosis (Fig. 1).

Further investigations established operable CTEPH with significant coronary stenosis in the left circumflex and right coronary arteries. Subsequently, he underwent pulmonary endarterectomy (PEA) and coronary artery bypass grafting (CABG) surgery. There were no intraoperative complications. The immediate postoperative period was uneventful. The patient was commenced on therapeutic subcutaneous enoxaparin 0.75 mg/kg on the second postoperative day (POD2) as per the standard PEA protocol at our institution. Aspirin was resumed as per CABG protocol.

The patient had a complex and prolonged postoperative stay due to acute generalised exanthematous pustulosis (AGEP), secondary to penicillin or enoxaparin allergy. Hence, he was switched from subcutaneous enoxaparin to fondaparinux. While recovering from AGEP, the patient complained of progressively worsening abdominal pain and severe pain radiating to his left lower limb on POD 25. He remained haemodynamically stable and with low-grade fever (temperature of 37.6 C, and oxygen saturation of 96% on 3 L of oxygen). On examination, his abdomen was distended, and he had tenderness bilaterally in the lower quadrants and suprapubic region. Bowel sounds were present. His laboratory data were significant for a haemoglobin drop (68 g/L from 98 g/L), increased creatinine (166 umol/L from 94 umol/L), and rising lactate levels (4.0 mmol/L). Computed tomography (CT) of his abdomen and pelvis was obtained, which showed a large left retroperitoneal haematoma, measuring 13.9 cm, involving the left psoas muscle. An actively bleeding vessel was demonstrated on the contrasted scan. Fondaparinux and aspirin were stopped.

The patient underwent a further CT angiography to localise the responsible vessel. However, no vessel could be identified for angioembolisation. He was managed conservatively with tranexamic acid for one week. He also received multiple blood transfusions. Subsequently, the bleeding settled, and he was restarted on a prophylactic dose of fondaparinux on POD 35. This was converted to 20 mg of Rivaroxaban once daily after a repeated CT scan revealed interval stability of the retroperitoneal haematoma.

## Discussion

Though spontaneous retroperitoneal hematoma results from rupture of retroperitoneal vessels, its aetiology is unclear [4]. In this case report, the patient was on anticoagulation after PEA and low-dose ASA after CABG. In a study by Dr Thistlethwaite and colleagues, PEA concomitantly with CABG, demonstrated comparable outcome with no observed increase in bleeding [5]. Postoperatively, the patient was started on anticoagulation in line with the PEA management guidelines. As per our institutional protocol, post-operative PEA patients typically initiate subcutaneous enoxaparin at a dosage of 40 mg on the first postoperative day. This dosage is then escalated to 0.75 mg/kg twice daily starting from the second postoperative day onwards. Following an assessment for potential bleeding and with ongoing discharge planning, patients are subsequently transitioned to oral anticoagulation therapy, which may consist of either warfarin or a new oral anticoagulation (NOAC).Bleeding into the retroperitoneal space is a severe complication of anticoagulation. The clinical manifestations of SRH vary from leg pain or paresis to hypovolemic shock [1].

The literature review indicates high mortality of up to 30% due to anticoagulant-related SRH [1, 6]. Our patient presented with a feeling of abdominal fullness and pain radiating down his left leg and an unexplained and persistent decrease in haemoglobin despite transfusion. This led us to suspect the diagnosis of SRH. The site of the pain and mass effect depends upon the location and amount of bleeding. Persistent bleeding can result in abdominal distension, anaemia, and even abdominal compartment syndrome. The pain in the legs or buttocks can be attributed to the compression of the lumbar plexus, particularly the femoral nerve. A prompt diagnosis is required to institute corrective measures. Multi-slice computed tomography (CT) and arteriography are the most valuable modalities for diagnosis. In our case, we stopped anticoagulation, and the patient underwent a CT scan to assess the size and location of the hematoma and the possibility of embolisation of the culprit vessel. No vessel could be identified for embolisation. The patient underwent serial CT scans to ascertain the change in size of the hematoma. May-Thurner Syndrome, a rare condition, occurs when the left common iliac vein (LCIV) is compressed by the right common iliac artery (RCIA). This compression can result in venous outflow obstruction, potentially leading to deep vein thrombosis (DVT) [7]. However, in this case, a CT scan did not reveal any compression of the LCIV as a contributing factor to the DVT. Though certain studies recommend reversal of anticoagulation, no reversal was performed [6]. During this period, he also received multiple blood transfusions to prevent significant anaemia.

Most studies suggest that though surgical management of hematoma is possible, it is associated with considerable morbidity and mortality. Percutaneous drainage may be attempted in case of worsening symptoms and progressive neurological deficits.[2] Nonetheless, the natural evolution of SRH results in spontaneous resolution. Most hemodynamically stable patients can be managed by fluid resuscitation, blood transfusions, and correction of coagulopathy [8]. In our patient, the hematoma remained stable, and anticoagulation was restarted subsequently. The pain and weakness in his leg resolved spontaneously, and we were able to discharge him satisfactorily.

## Conclusion

Elderly patients in intensive care and on anticoagulation have a significant risk of developing a spontaneous retroperitoneal hematoma, which is suggested by abdominal distension and lower limb weakness. There should be a low threshold for scanning them. To avoid further complications, conservative management is recommended.

## Data Availability

Not applicable.
